# Zinc Status in South Asian Populations—An Update

**DOI:** 10.3329/jhpn.v31i2.16378

**Published:** 2013-06

**Authors:** Saeed Akhtar

**Affiliations:** Department of Food Science and Technology, Bahauddin Zakariya University, Multan, Pakistan

**Keywords:** Growth, Infection, Malnutrition, Zinc, South Asia

## Abstract

This article attempts to highlight the prevalence of zinc deficiency and its health and economic consequences in South Asian developing countries and to shed light on possible approaches to combating zinc deficiency. A computer-based search was performed on PubMed, Google, and ScienceDirect.com to retrieve relevant scientific literature published between 2000 and 2012. The search yielded 194 articles, of which 71 were culled. Studies were further screened on the basis of population groups, age and sex, pregnancy, and lactation. The most relevant articles were included in the review. Cutoffs for serum zinc concentration defined for zinc deficiency were 65 µg/dL for males and females aged <10 years, 66 µg/dL for non-pregnant females, and 70 µg/dL for males aged ≥10 years. Population segments from rural and urban areas of South Asian developing countries were included in the analysis. They comprised pregnant and lactating women, preschool and school children. The analysis reveals that zinc deficiency is high among children, pregnant and lactating women in India, Pakistan, Bangladesh, Sri Lanka, and Nepal. Diarrhoea has been established as a leading cause to intensify zinc deficiency in Bangladesh. Little has been done in Sri Lanka and Nepal to estimate the prevalence of zinc deficiency precisely. A substantial population segment of the South Asian developing countries is predisposed to zinc deficiency which is further provoked by increased requirements for zinc under certain physiological conditions. Supplementation, fortification, and dietary diversification are the most viable strategies to enhancing zinc status among various population groups.

## INTRODUCTION

Zinc plays a critical role in normal functioning of body and is integrated with several enzyme systems. Gene expression, cell division, immunity, and reproduction are important biological functions of zinc (1). Adequate dietary intake of zinc has been shown to exert ameliorating effect on the skin, and this attenuates the likelihood of restricted linear growth in young children. Neurobehavioural disturbances among infants, hypoguesia, the chronic non-healing leg ulcers and repeated infections are common among zinc-deficient subjects of all ages. Pregnant women with zinc deficiency are at the risk of complicated pregnancy outcomes (2-4).

Predictably, half of the global population is at the risk of low intakes of zinc. Around 800,000 deaths among children below 5 years of age occur annually due to diarrhoea (176,000), pneumonia (406,000), and malaria (207,000). High mortality rate among children resulting from these infections has been reported to be associated with inadequate zinc intake. The loss incurred due to zinc deficiency amounts to nearly 16 million global disability-adjusted life years (DALYs) (5-7).

Prevalence of zinc deficiency in developing countries is very common, and 61% of the population is at an increased risk of low dietary zinc intake (8). Almost 4% of child mortality and DALYs have been associated with zinc deficiency (5). Studies demonstrated that 49.4% of adolescent girls in Delhi (9) and 52% non-pregnant women of central India (10,11) suffered from zinc deficiency. There is a paucity of literature to reflect zinc deficiency in Pakistan. However, limited studies reported 54.2% children and 37.1% preschool children to be zinc-deficient (12,13). Malnutrition and poverty in Bangladesh are the leading causes of high rate of zinc deficiency, especially at early pregnancies (14). Similarly, zinc deficiency is prevalent among various population groups in Sri Lanka, especially among preschool children and adolescents (15). Prevalence of zinc deficiency among non-pregnant Nepalese women was attributed to excessive use of phytate-rich foods with limited dietary zinc intakes (16).

Although zinc was omitted from micronutrient priority list of the United Nations in 1999, compelling evidence exists to suggest a global realization on health benefits of zinc nutrition in the last decade. Zinc deficiency, as a public-health problem, is now recognized among the South Asian developing economies. Despite this recent recognition, rigorous efforts are not underway to reduce the gravity of the problem. Currently, no precise data on the extent of prevalence of zinc deficiency are available at the national level; hence, it is the proper time to assess the severity of the risk of zinc deficiency in South Asian populations. Although several international organizations, such as International Zinc Nutrition Consultative Group (IZiNCG), Micronutrient Initiatives (MI), Food and Agriculture Organization (FAO), World Health Organization (WHO), Global Alliance for Improved Nutrition (GAIN), Sharing United States Technology to Aid in the Improvement of Nutrition (SUSTAIN), and United Nations International Children's Emergency Fund (UNICEF), have been perpetually working in the developing countries to improve nutritional status of the vulnerable populations, the outcome of these efforts has not yet knocked any mitigating effect on the prevalence of zinc deficiency among the predisposed population groups. A plausible explanation for inefficacy of these efforts is the prevalence of poverty, illiteracy, lack of infrastructure, and government priorities to eliminate hidden hunger in the region.

This review highlights current situation of zinc deficiency in South Asian developing countries and examines causes of zinc deficiency, adverse health effects, and possible intervention strategies in India, Pakistan, Bangladesh, Sri Lanka, and Nepal. Future directions have been provided to delineate the action-oriented strategies and plans that need to acquire momentum for virtual elimination of zinc deficiency in this region.

### Zinc deficiency in South Asian developing countries

Sufficient data are available to estimate the extent of zinc deficiency in South Asian developing countries. Data obtained through national nutrition surveys, small representative epidemiological studies, complete randomized trials (CRTs), and meta-analyses illustrate the severity of zinc deficiency in South Asian societies. A plethora of publications in the literature is, however, available to address issues relating to zinc nutrition in westernized world, and two complete reviews (1,17) gained wider recognition worldwide to establish zinc deficiency as a public-health problem. Amongst several other instrumental determinants, low dietary intake, low bioavailability, unhygienic conditions, recurrent infections, poor socioeconomic status, unstable political situation, improper priority setting in national agenda, inappropriate monitoring, inadequate surveillance, and lack of public awareness have been identified as leading causes of high prevalence of zinc deficiency in low-income South Asian countries.

#### Zinc deficiency in India

India, with its population of 1.2 billion, is ranked 2^nd^ in the world for the number of children (47%) suffering from malnutrition and 15^th^ amongst leading countries with hunger situation. About 2.1 million deaths among Indian children (<5 years) occur every year because of diarrhoea, typhoid, malaria, measles, and pneumonia. Evidently, 1,000 children die each day for diarrhoea alone (18,19).

Reliable national-level data are scant to clearly demonstrate the extent of the prevalence of zinc deficiency. However, numerous small studies highlight the likelihood of widespread marginal zinc deficiency among children and pregnant women in India. Recent epidemiological studies have confirmed high prevalence of zinc deficiency among children belonging to low socioeconomic groups in five major Indian states, reporting an overall zinc deficiency of 43.8% (cutoff level ≤65 μg/dL), with the highest in Orissa (51.3%), followed by Uttar Pradesh (48.1%), Gujarat (44.2%), Madhya Pradesh (38.9%), and Karnataka (36.2%). Table 1 indicates the distribution of children based on serum zinc levels of <55 μg/dL, <60 μg/dL, and <65 μg/dL (11,20). One more recent cross-sectional study (n=630) confirmed low plasma zinc concentration and poor cognitive performance in 45% of the adolescent girls in India, signifying the need to adopt dietary zinc intake for normal health (21). Nearly 64.6% of the pregnant women have been shown to suffer from zinc deficiency in the state of Haryana because of low dietary intake of zinc. Similarly, a high prevalence of zinc deficiency (41%) has been reported among the nulliparous non-pregnant women in India (22,23). Current zinc status of various population groups in India warrants the need to undertake multicentre studies, more rigorous analyses, and extensive surveys to identify the factors leading to zinc deficiency. More efforts are needed to estimate the magnitude of zinc deficiency among population groups and to devise strategies to combating this nutritional problem in India.

**Table 1.** Distribution of children below five years of age in India according to their serum zinc levels

State	Serum zinc levels		
	<55 μg/dL	<60 μg/dL	<65 μg/dL
Gujarat (n=353)	25.8	34.0	44.2
Karnataka (n=356)	19.1	26.4	36.2
Madhya Pradesh (n=285)	14.7	22.8	38.9
Orissa (n=345)	34.5	43.2	51.3
Uttar Pradesh (n=316)	29.4	40.2	48.1
Total (n=1655)	25.0	33.5	43.8

Derived from Kapil and Jain 2011 11

#### Zinc deficiency in Pakistan

Estimates indicate infant and maternal mortality rates to be 73 per 1,000 livebirths (24) and 276 per 100,000 livebirths (25) respectively in Pakistan. Several studies revealed zinc deficiency to be prevalent among schoolgoing children (54.2%) and preschool children (37.1%) in Pakistan. Pregnant women (54%) were also shown to suffer from zinc deficiency in Sindh province of Pakistan, and 50% of the participating subjects in another study manifested low plasma levels of zinc, suggesting marginal zinc deficiency among Pakistani population (12,13). Prevalence of zinc deficiency in pregnant and non-pregnant women in Pakistan has been presented in Table 2 (26).

Diarrhoea, pneumonia, and malaria are closely associated with zinc deficiency (27,28); therefore, zinc supplementation and fortification have been recommended as potential approaches to combating zinc deficiency in Pakistan (29). Nutritional programmes for preventive zinc supplementation for children below 5 years of age have been suggested for inclusion in the national strategies to reducing zinc deficiency (30). Moreover, addition of zinc fortificants in wheat flour—the main dietary staple of the Pakistani population—is a focus of the policy-makers and flour industry to control zinc deficiency in the country (31). However, such addition of fortificants in wheat flour needs to be critically evaluated for the forms of fortificants and fortification levels to ensure the undamaging health effects of these compounds (32).

#### Zinc deficiency in Sri Lanka

There have been fewer reports on magnitude of the prevalence of zinc deficiency in Sri Lanka. Reports to assess the gravity of prevalence of zinc deficiency at the national level are not available for Sri Lanka. However, several small studies (33,34) confirmed low serum zinc concentrations (cutoff serum zinc <65 µg/dL) in Sri Lankan populations. Nearly 57.0% male and 50.0% female preschool children were found to be zinc-deficient. Information derived from 24-hour dietary recall of these children validated that their zinc intake was only half of the RDA due to inadequate consumption of foods of animal origin (34). Another study demonstrated that 51.5% of adolescent males and 58.3% females were affected by zinc deficiency (serum zinc <65.032 µg/dL) (35). Low intakes of foods of animal origin and low bioavailability of zinc in plant-based diets have been attributed to high prevalence of zinc deficiency.

**Table 2.** Zinc deficiency in pregnant and non-pregnant women in Pakistan (2011)

Area	Non-pregnant women			Pregnant women		
	Deficient (<60 µg/dL)	Non-deficient (≥60 µg/dL)	N	Deficient (<60 µg/dL)	Non-deficient (≥60 µg/dL)	N
Total	41.6	58.4	5,953	48.3	51.7	791
Urban	38.2	61.8	2,395	47.2	52.8	285
Rural	43.2	56.8	3,558	48.7	51.3	506

Derived from National Nutrition Survey Report (NNS) 26; N=Total no. of subjects

#### Zinc deficiency in Bangladesh

A recent study showed 41%, 35%, and 18% of the total subjects under study to be stunted, underweight, and wasted respectively while 16%, 11.5%, and 3% were severely stunted, underweight, and wasted respectively in rural Bangladesh (36). Primarily, two fundamental indicators are used in estimating the prevalence of zinc deficiency among vulnerable population groups, i.e. stunting and inadequacy of zinc intake. Higher stunting rate (43%) was noted among children below 5 years of age, suggesting zinc deficiency to be extensively prevalent in Bangladesh (5). Zinc deficiency is more existent in communities consuming restricted meat-based diets and increased amount of vegetables containing more phytates.

Diarrhoea is regarded a fundamental cause of child deaths in Bangladesh, and zinc supplementation can reduce morbidity and mortality by preventing 30,000 to 75,000 childhood deaths (14,37,38). Another study elucidated that a 5-day course of zinc treatment exerted the same influence as that of a 10-day course to prevent diarrhoea over the 3 months following treatment of a diarrhoeal episode (Table 3) (39). Zinc supplementation resulted in 15% less incidence of diarrhoea, thus saving the lives of 30,000-75,000 children per year. A reduction of 15% in the illness duration and 16% reduction in the likelihood of disease progression were observed as a positive effect of zinc supplementation in Bangladesh (40,41).

**Table 3.** Characteristics of the study participants who received either a 5-day or a 10-day zinc treatment during acute diarrhoeal episode

Characteristics	Zinc treatment group	
	5 days (N=803)	10 days (N=819)
Age (months)	25.6±16.2	25.6 ± 16.2
z-scores		
Weight-for-height	-0.73±1.0	-0.66±1.0
Height-for-age	-2.17±1.2	-2.11±1.1
Weight-for-age	-1.78±1.0	-1.68±1.0
Maternal education (years)	5.8±2.7	6.1±2.7
Family-size (persons)	6.1±2.3	6.0±2.2
Sex (male) (%)	50.8	48.7
Tubewell (drinking-water) (%)	98.4	97.9
Sanitary latrine (%)	21.8	19.7

Derived from Alam *et al.* 2011 39; Values are means±SD or percentage

Several rudimentary issues have been identified that hamper the accomplishment of challenging task of promoting better nutrition in Bangladesh. Government priority to scale up sustainable micronutrient interventions and capacity-building at local and national levels to implement these interventions are currently lacking and require a momentous leap to address this nutritional issue. Moreover, there is a dire need to chalk out plans and policies to curb poverty through the promotion of entrepreneurship, agriculture-based cottage industry, and fisheries in the region.

#### Zinc deficiency in Nepal

Nepal, as one of the world's poorest countries and the poorest in South Asia, witnesses infant mortality to be 96 per 1,000 livebirths. Poverty remains a substantial impediment to ensure the supply of sufficient balanced food to the population. Around 500 non-pregnant Nepalese women, examined for plasma zinc concentration, exhibited elevated zinc deficiency. Zinc deficiency was more common among women of childbearing age. Similarly, zinc deficiency has been shown to be the major cause of poor newborn's health and poor host defense against infections in Nepal (16). Pregnant women in Nepal are more vulnerable to multiple micronutrient deficiencies. Among 1,165 pregnant women in rural Nepal, 61% demonstrated higher prevalence of micronutrient deficiencies, including that for zinc (42).

Beneficial effects of zinc supplementation have been seen on child growth, particularly among vulnerable groups (43). Moreover, multiple micronutrients reduced the burden of zinc deficiency in rural Nepal (44). By contrast with the foregoing results indicating beneficial effects of micronutrient supplementation, daily supplementation to young children with zinc alone and/or with other micronutrients in Southern Nepal depicted no effect on mortality rate among infants. However, they had a protective effect against diarrhoea, dysentery, and acute respiratory illness (45,46). Likewise, zinc and multiple micronutrients did not indicate any additional beneficial effect on improving maternal haematologic status during pregnancy (47,48).

Apparently, the existing infrastructure and health service system in Nepal need to be completely revamped to ensure safe and nutritious food supply to the population. This seems much challenging in the absence of good governance, international support, and development of awareness among the stakeholders.

### Aetiology of zinc deficiency

Zinc nutrition had been a neglected area in developing countries; nevertheless, a wave of realization on the health significance of zinc spurred in early 2000, and investigations were initiated to explore the causes of zinc deficiency. Extent of the prevalence of stunting and inadequacy of zinc in the diets had been the markers of estimating zinc deficiency (1,49). Several studies confirmed inadequate intake of zinc to be one of the most significant determinants for the development of zinc deficiency (50). Amongst other leading factors that develop zinc deficiency, elevated zinc requirements, poor absorption, increased losses, and impaired utilization of zinc by the body are also important in playing a vital role. A strong association between the consumption of plant-based diets and zinc deficiency in developing countries has been widely reported (51). High phytic acid content in Asian diets, especially cereals and cereal-based diets, forms zinc-phytic acid complexes in the intestine (52) and markedly inhibits intestinal absorption of zinc (53,54). Bioavailability of zinc is greatly influenced in the presence of several other inhibitors (53), including calcium and polyphenols (55). Table 4 presents the physiologic requirements for absorbed zinc during childhood by age-group and sex and during pregnancy and lactation (56).

Decreased tendency among mothers for breastfeeding normally limits an optimal zinc supply to the infants. Similarly, need for zinc during pregnancy increases, especially among women with low plasma zinc, to meet foetal and maternal requirements (57). One group of researchers explicated that the requirement for zinc is boosted up during the early months of pregnancy. Therefore, zinc absorption is likely to increase at this stage (58). This condition is aggravated in instances where recurrent reproductive cycling takes place, and mothers are highly disposed to zinc deficiency (59).

**Table 4.** Estimated physiologic requirements for absorbed zinc during childhood by age-group and sex and during pregnancy and lactation

Age, sex, stage	Reference weight (kg)	Physiologic requirement (mg/day)
6-<12 months	9	0.84
1-<3 years	12	0.83
3-<6 years	17	0.97
6-10 years	25	1.12
10-12 years, M	35	1.40
10-12 years, F	37	1.26
12-15 years, M	48	1.82
12-15 years, F	48	1.55
15-18 years, M	64	1.97
15-18 years, F	55	1.54
At pregnancy	-	2.27
During lactation	-	2.89

Derived from WHO 1996 56

Sufficient body of literature supports diarrhoea as a potent cause of zinc deficiency among population groups living in developing countries. The risk of zinc deficiency multiplies during diarrhoea and is associated with gross losses of zinc through the faeces (60,61). Zinc has been found to have a therapeutic benefit in acute and persistent diarrhoea and may also reduce infant mortality (62,63).

### Health and economic consequences of zinc deficiency

**S**higellosis, Campylobacteriosis, *E. coli* infection, *Staphylococcus aureus* infection, salmonellosis, listeriosis, and cholera have been shown to heighten the disease burden and exert a damaging effect on human health in underdeveloped communities (64,65). Widely-reported incidences of such infections, especially among children in developing world, are not the direct outcomes of zinc deficiency. However, the likelihood of these incidences is reduced to certain extent through zinc therapy (66). There is mounting evidence to support the role of zinc in linear growth and weight gain (67). Premature delivery and low birthweight of babies have been the known outcomes of zinc deficiency in mothers, leading to restricted zinc supply to the foetus (2). In total, an estimate has confirmed 800,000 childhood deaths annually due to zinc deficiency. These deaths and increased morbidity are associated with infectious diseases, resulting in 1.9% of global DALYs. Another group of researchers explored high mortality rate among children, signifying 10.8 million deaths annually in the world. Predominant health outcomes of zinc deficiency include short stature, impaired immune function, and other disorders, like respiratory infections, malaria, and diarrhoeal diseases (68,69).

Zinc deficiency exerts greater influence on mortality rates, causing 2.7% of global DALYs compared to around 6% caused by iron deficiency. Low- and middle-income developing countries are the largest victims of zinc deficiency as it negatively impacts the health and leads to disabilities, such as cognitive impairment and decreased work-capacity (70).

### Current strategies to combating zinc deficiency

Supplementation, fortification, and dietary diversification are the most viable strategies to enhancing zinc status among various population groups. To implement these strategies successfully, a thorough knowledge of food constituents, chemical composition of supplements or fortificants and their bioavailability is considerably important (71). It seems more practicable to practise supplementation by integrating it with ongoing national programmes for health and nutrition. These approaches can be more effective among population groups with increased awareness on the deleterious health effects of zinc and related sequelae. The strategies must also be well-coordinated among government, education, public health and industry sectors, international organizations, and consumer groups.

#### Supplementation

Zinc supplementation has been widely practised in the majority of South Asian poorer economies. However, there is a significant heterogeneity in the results of various studies. This strategy holds certain advantages over others, like rapid improvement in zinc status, easy administration, individual compliance, and being equally applicable for all age-groups (1). Viability of zinc supplementation programmes in developing countries, as a defensive approach against diarrhoea, pneumonia, respiratory tract infection, restricted growth, and mortality, is widely-documented in the literature. Several studies demonstrated an increase in linear growth and weight gain attributable to zinc supplementation among children in developing countries (72). Zinc supplementation has been extensively employed in Bangladesh, and dramatic reductions in the occurrence of persistent diarrhoea and severe acute lower respiratory infections were witnessed (73-75). In reality, no planned national-level zinc supplementation programmes have been launched in most of the South Asian developing countries. Huge investments, economic and social reforms, poverty alleviation, and coordinated awareness programmes are significant determinants to shape nations’ future in terms of health and economic well-being of the populations.

#### Food fortification

Food fortification has been widely-debated as a potential approach to combating micronutrient deficiencies in developing countries. Seemingly, there is a great diversity for the success and failure of this strategy in the developing world. South Asian low-income countries are still far away to reap the fruit of this strategy as poverty has been the most significant determinant of failure. There are several other underlying determinants that impact the successful implementation of fortification programmes critically in these regions, including government commitment, legislation, education, awareness, and cost of fortification. Zinc fortification in South Asian developing countries is not yet well-structured despite the fact that it showed a positive effect on total zinc absorption and enhanced zinc status in populations (2,76).

According to World Health Organization, food fortification is a viable approach in lower-income countries, and its impact on target population's nutrient intake is promising (77). Cereals and cereal products are the commonly-consumed foods in South Asian developing countries. Therefore, zinc fortification in cereal products would have a positive impact on serum zinc concentration (78).

There are several technical considerations for zinc fortification programmes, including selection of the food vehicle(s), selection of the form of zinc fortificants, determining the level of zinc fortificants, and cost of fortification programmes (32).

Biofortification is another approach to curbing micronutrient deficiencies in developing countries. This intervention involves agricultural, agronomic, or genetic means to boost up the level of particular micronutrient in the crop. Currently, this technology is being focused to elevate the zinc, iron, and pro-vitamin A carotenoid levels in some of the world's most important staple food-crops, i.e. rice, wheat, and maize (79).

#### Dietary diversification

Emphasis has been laid down in the literature to adopt food dietary diversification as a preventive strategy to curtail the gravity of micronutrients deficiency. Dietary diversification employs consumption of meat, poultry or fish, and all good sources of readily-available zinc (80). Selection of foods, modification in traditional methods of food preparation and focused food production are a few important considerations for adequate zinc supply through this approach. For example, phytate contents in cereals and legumes would be reduced by enzymatic hydrolysis of phytic acid and by germination and fermentation process. Similarly, antinutrients, such as saponins and polyphenols, can also be minimized through soaking for enhanced zinc absorption (53,81). Furthermore, this approach is particularly beneficial to improve bioavailability of iron, vitamin B_12_, vitamin A, and calcium (82). This strategy holds a great potential for being economical, sustainable, and culturally acceptable in developing countries.

### Future directions

Zinc deficiency is now a public-health problem, especially in South Asian developing economies. Apparently, no policy seems to exist for combating the issue and reaching an equitable and sustainable solution. Similarly, no strategy would be successful in the absence of reliable data, precisely showing the magnitude of the prevalence of zinc deficiency in South Asia. In most of the studies and surveys carried out so far in the developing countries to gauge the zinc status, the sample-sizes had been too small to yield results that could be applied to the whole population. Thus, the nutrition survey mechanism needs to be revitalized to cover the whole population for consistent results. Several previous efforts to elevate zinc status among vulnerable groups revealed that the communities did not show tendency to receive the supplementation, rather the conventional pharmacological treatment for rapid relief was considered more acceptable. Therefore, creating awareness through all means and sources would have an encouraging impact in reducing zinc deficiency in the poorer settings. Compelling scientific evidence suggests a number of approaches to making supplementation programmes successful. These include combining supplementation with oral rehydration salt (ORS) for the management of diarrhoea, preparation of appropriate zinc formulations, training/education, consistent availability of zinc products, and monitoring and evaluation. Industry, governments, donors, and consumers are the major drivers for the success of national nutrition programmes. Additionally, targeted supplementation, fortification, and dietary strategies need to be focused to alleviate the likelihood of zinc deficiency in South Asian developing countries. This needs to be associated with strong advocacy, political commitment, a precise infrastructure, financial investment, and capacity-building to implement such programmes. Evidently, more studies in developing countries are needed to evaluate the current nutritional status of the populations critically and to achieve the Millennium Development Goals for health, the core idea of which is to improve maternal and child health.

### Conclusions

Zinc deficiency in South Asian developing countries is considerably prevalent. In retrospect, Bangladesh seems to have the highest prevalence of zinc deficiency. Populations from India, Nepal, Sri Lanka, and Pakistan are also affected by zinc deficiency. Inadequate intake of zinc has been regarded as one of the most significant causes of zinc deficiency. Diarrhoea, respiratory infections, and malaria have been associated with zinc deficiency, particularly in low-income developing countries. Supplementation, fortification, and dietary diversification are the most viable strategies to enhancing the zinc status among various population groups. Food fortification is increasingly recognized as an effective approach to improving a population's micronutrient status. Dietary diversification employs consumption of meat, poultry or fish, and all good sources of readily-available zinc and is considered a potential preventive approach to reducing zinc deficiency. Zinc supplementation has been widely practised in majority of these countries. These strategies need to be harmonized in a sociocultural perspective and must be coordinated in developing countries at the national level. Evidently, development of awareness among the vulnerable populations has shown promise to mitigate the devastating impact of this nutritional deficiency.
